# Discovery of a Novel Mutation (X8Del) Resulting in an 8-bp Deletion in the Hepatitis B Virus X Gene Associated with Occult Infection in Korean Vaccinated Individuals

**DOI:** 10.1371/journal.pone.0139551

**Published:** 2015-10-05

**Authors:** Hong Kim, Jeong-Ryeol Gong, Seoung-Ae Lee, Bum-Joon Kim

**Affiliations:** Department of Microbiology and Immunology, Liver Research Institute, Cancer Research Institute and SNUMRC, College of Medicine, Seoul National University, Seoul, Korea; Academia Sinica, TAIWAN

## Abstract

Universal infantile hepatitis B virus (HBV) vaccination may lead to an increase in vaccine escape variants, which may pose a threat to the long-term success of massive vaccination. To determine the prevalence of occult infections in Korean vaccinated individuals, 87 vaccinated subjects were screened for the presence of HBV DNA using both the nested PCR protocol and the VERSANT HBV DNA 3.0 assay. The mutation patterns of variants were analyzed in full-length HBV genome sequences. Their HBsAg secretion and replication capacities were investigated using both *in vitro* transient transfection and *in vivo* hydrodynamic injection. The presence of HBV DNA was confirmed in 6 subjects (6.9%). All six variants had a common mutation type (X8Del) composed of an 8-bp deletion in the C-terminal region of the HBV X gene (HBxAg). Our *in vitro* and *in vivo* analyses using the full-length HBV genome indicated that the X8Del HBxAg variant reduced the secretion of HBsAg and HBV virions compared to the wild type. In conclusion, our data suggest that a novel mutation (X8Del) may contribute to occult HBV infection in Korean vaccinated individuals via a reduced secretion of HBsAg and virions, possibly by compromising HBxAg’s transacting capacity.

## Introduction

Hepatitis B virus (HBV) infection is a global health problem, and more than 350 million people are chronic carriers of the virus [1]. The infection is associated with a large spectrum of clinical manifestations, ranging from acute or fulminant hepatitis to various forms of chronic infection, including asymptomatic carriers, chronic hepatitis, cirrhosis, and hepatocellular carcinoma (HCC). The yearly number of deaths caused by HBV-related diseases has been estimated to be approximately 600,000 worldwide [2].

Because HBV is most commonly vertically transmitted from mother to child at birth or during early childhood in highly endemic areas [3–5], universal infantile vaccination is the most powerful method for preventing HBV transmission. South Korea is recognized as an endemic area for HBV infection; based on the Korean National Health and Nutrition Survey of 2012, the prevalence of HBsAg was 4.2% in men and 3.1% in women [6]. HBV vaccination was first introduced into the Korean population in 1983 [7] and dramatically reduced the prevalence of HBsAg-positive chronic carriers from more than 10% to 3.7% in 2012 over a period of approximately 30 years [6]. However, immune pressure by this successful vaccination may lead to the generation of diverse mutation patterns capable of immune evasion among the vaccinated population [8–10]. An extraordinary prevalence of genotype C2 was also reported in South Korea; this genotype is more prone to mutations and is associated with more severe liver diseases and lower antiviral responses compared to genotype B [11,12]. Thus, a high prevalence of basal core promoter (BCP) double mutations and the presence of distinct immune responses against HBV proteins in the Korean population may lead to the generation of HBV variants that are never or rarely encountered in other areas, resulting in unique clinical manifestations in Korean chronic patients [13–30].

Recently, we demonstrated that several types of naturally occurring mutations in the preS and S regions, which are related to clinical severity in Korean chronic patients (i.e., the preS1 start codon deletion, preS2 deletion, W4P/R in preS1, and sW182*in S), were also frequently found in occult subjects [26]. These results strongly supported the hypothesis that these mutations were horizontally transmitted from Korean chronic patients to the HBsAg-negative Korean population. Furthermore, occult infection-related HBsAg variants in South Korea led to a lower secretion capacity of HBsAg, causing false-negative results in commercial screening kits [31]. Thus, there is potential for the presence of distinct vaccine escape mutations among the vaccinated population in South Korea.

The aim of this study was to investigate the prevalence and mutation patterns of occult HBV infection in a Korean vaccinated cohort and to elucidate the molecular mechanism underlying occult infection or vaccine escape.

## Materials and Methods

### Study Subjects

**Fig 1:**
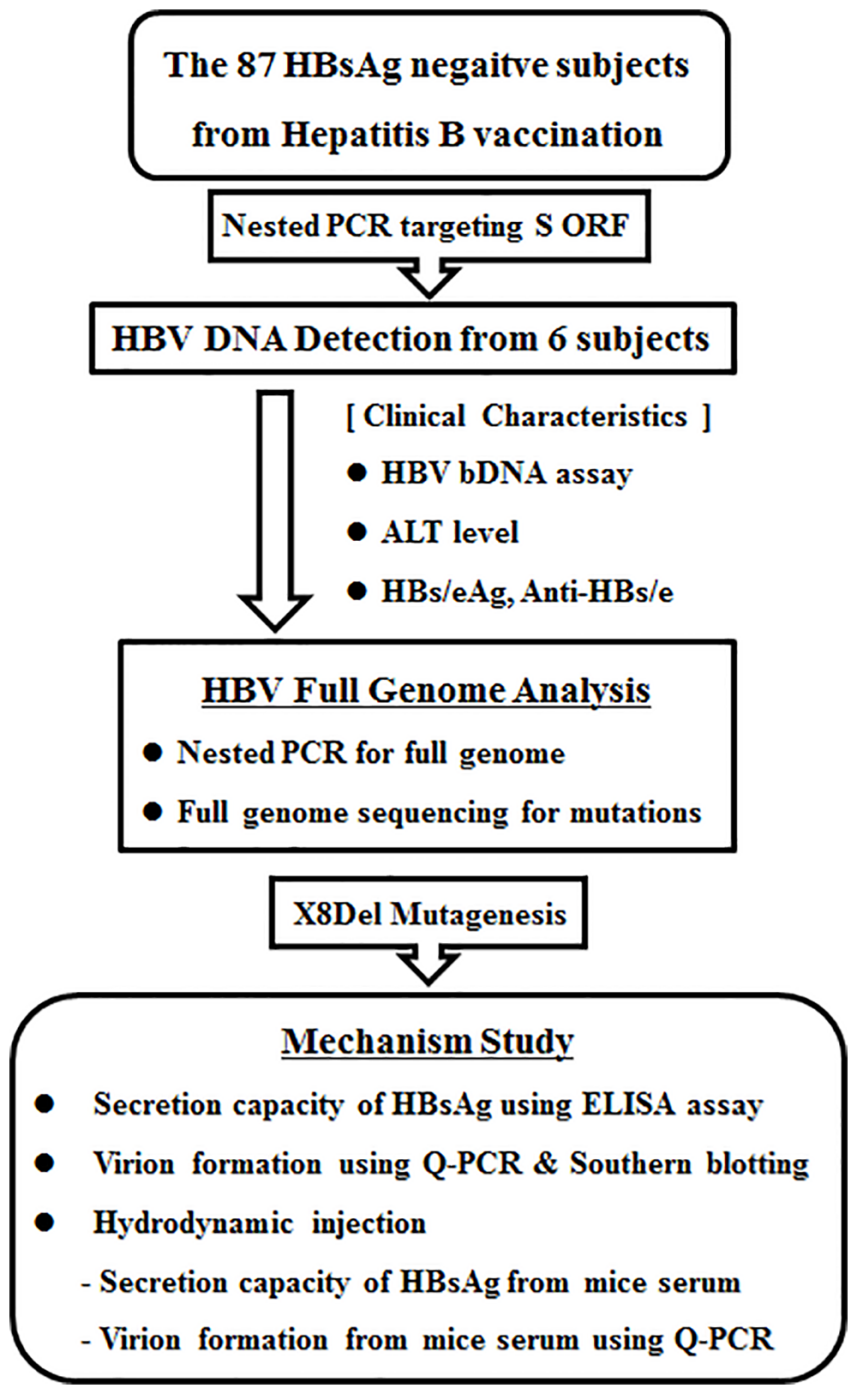
Strategy of the current study for the elucidation of molecular epidemiologic features and the mechanism underlying a novel X8Del variant that lead to occult infections in Korean vaccinated individuals.

Among the three hundred patients who visited Sung-Shim local hospital (a center for primary care) in Seoul, South Korea, in 2005, a total of 87 subjects with no clinical liver disease who had received the hepatitis B vaccine (HepaVaxR, Green Cross, Seoul, Korea) were selected for this study. The level of HBV surface antigen (HBsAg) detection was assessed in the 87 subjects using the ARCHITECT i2000 System (Abbott, Chicago, IL, USA). In 6 subjects exhibiting PCR amplification according to a nested PCR protocol, the levels of alanine aminotransferase (ALT), hepatitis B e antigen (HBeAg), anti-hepatitis B e (anti-HBe) and anti-HBs were also measured with the ARCHITECT i2000 System (Abbott, Chicago, IL, USA). HBV-bDNA from the 6 hepatitis B vaccinated subjects was investigated using the Versant HBV DNA Assay version 3.0 (bDNA, Siemens, New York, NY, USA). All work was approved by the institutional review board of Seoul National University Hospital (IRB No. C-1404-070-572). The experiment was mainly based on the virion DNA extracted from isolates; therefore the research was done without informed consent and a waiver of informed consent was agreed upon by the IRB. The flowchart and research strategy used in this study are presented in Fig 1.

### HBV DNA Extraction and PCR Amplification

HBV DNA was extracted from 200 μl of serum using the QIAamp DNA Blood Mini Kit (QIAGEN, Hilden, Germany). To elucidate the prevalence of vaccine escape variants, we applied nested PCR protocols producing 1378-bp coverage of the entire S ORF to all 87 Korean vaccinated HBsAg-negative subjects as previously described [26]. Briefly, the first round of PCR was performed using the sense primer PreS2-Del-F2 (GenBank accession number AF286594, nt positions 2814–2832, 5′-GGG TCA CCA TAT TCT TGG G-3′) and the antisense primer HB2R (nt positions 1029–1049, 5′-CAT ACT TTC CAA TCA ATA GG-3′), which target a large surface region. The second round of amplification was performed using the sense primer Del-PRA-F1 (nt positions 2887–2907, 5′-CTT GGG AAC AAG AGC TAC AGC-3′) and the HB2R primer.

### HBV Full-Length Genome Analysis

The nested PCR protocol for the HBV full-length genome sequencing analysis was performed using a modified version of a previously described method [13,17]. Briefly, the first round of amplification was performed using the sense primer HBV-Nor-Full-F (GenBank accession number AY641558, nt positions 658–681, 5'-CCG TTT CTC CTG GCT CAG TTT ACT-3') and the antisense primer HBV-Nor-Full-R (nt positions 635–658, 5'-GAC TGA GGC CCA CTC CCA TAG GAA-3'), which amplify the full-length HBV genome (approximately 3.2 kbp). The second round of PCR was performed with eleven primer sets using the i-MAX II DNA polymerase (iNtRON Bio, Seoul, Korea) in a 20 μl mixture. The sequences of the eleven primer sets are shown in S1 Table. For direct sequence analysis, an Applied Biosystems model 373A automatic sequencer and a BigDye Terminator Cycle Sequencing kit (Perkin-Elmer Applied Biosystems, Norwalk, CT, USA) were used for the sequencing. The obtained sequences were aligned with the sequences of eight HBV references (GenBank accession numbers M57663, AB100695, AB074755, AY247032, AY641558, X02496, AB106564, and X75663) using the multiple-alignment algorithm in the MegAlign package (DNASTAR, Madison, WI, USA). The phylogenetic tree was constructed using the neighbor-joining method in the MEGA4 program (Molecular Evolutionary Genetic Analysis, AZ, USA). The percentages indicated at the nodes represent bootstrap levels supported by 1,000 re-sampled data sets. Bootstrap values < 50% are not shown. Numbers in parentheses are GenBank accession numbers.

### Generation of the HBV Full-Length Genome Construct with the X8Del Mutation using Site-Directed Mutagenesis

A mutant HBV full-length genome construct with an eight-nucleotide deletion in HBxAg (hereafter, pHBV1.2X-X8Del) was generated by site-directed mutagenesis of the wild-type pHBV-1.2x vector (hereafter, pHBV1.2X-Wild) (Genotype C, GenBank No. AY641558), which was kindly provided by Prof. Jung G, *et al* [32]. The mutagenesis was performed using the forward primer X8D-F (nt position 1743–1789, 5’-GGG GAG GAG ATT AGG TTA ATG ATC T**tt gta cta** GGA GGC TGT AGG CA-3’) and reverse primer X8D-R (nt position 1743–1789, 5’-TGC CTA CAG CCT CC**t agt aca a**AG ATC ATT AAC CTA ATC TCC TCC CC-3’).

### Cell Culture and Transfection

HuH-7.5 cells were cultured in Dulbecco’s modified Eagle’s medium (HyClone, Thermo Scientific, Waltham, MA, USA) containing 10% fetal bovine serum (GibcoBRL, Grand Island, NY, USA) and 100 μg/ml of penicillin-streptomycin (GibcoBRL, Grand Island, NY, USA). To measure the capacity for HBsAg secretion and virion formation, 4 μg each of the pHBV1.2X-Wild and pHBV1.2X- X8Del constructs were transiently transfected into HuH-7.5 cells using Lipofectamine2000 (Invitrogen, Carlsbad, CA, USA) in six-well plates (2 x 105 cells/well). The cells were incubated at 37°C with 5% CO2 for 48 hours. Next, the supernatant was centrifuged at 1,300 rpm for 3 minutes to remove the cell debris and stored at -70°C. The pellets were washed twice with phosphate-buffered saline (PBS) (GibcoBRL, Grand Island, NY, USA) and lysed with mild Reporter Lysis Buffer (RLB) (Promega, Fitchburg, WI, USA).

### HBsAg ELISA Assay

To compare the secretion capacity between pHBV1.2X-Wild and pHBV1.2X-X8Del from the supernatants and lysed pellets, an HBsAg ELISA was conducted using the commercial Bioelisa HBsAg color ELISA Kit (BIOKIT, Barcelona, Spain) according to the provided experimental method. To normalize HBsAg in the transfected cells, the expression level of β-galactosidase was also measured using a β-galactosidase enzyme assay system kit (Promega, Fitchburg, WI, USA).

### Q-PCR Analysis

After the culture medium was centrifuged to remove the cellular debris, the supernatant underwent ultracentrifugation at 20,000 rpm in an SW28 rotor (Beckman Coulter, USA) for 2 hours at 4°C. The collected cell pellet was washed and resuspended with PBS, and viral DNA was extracted using the QIAamp DNA Mini Kit (QIAGEN, Hilden, Germany) according to the manufacturer’s instructions. HBV DNA replication was measured using a quantitative real-time PCR (Q-PCR) targeting the HBV virion both in the supernatant and the pellet from the transfected cells. The PCR amplification was performed with a set of real-time PCR primers targeting the small S gene designed to amplify a 101-bp product with primer sequences as follows: sense primer Real-SF (positions 218–240, 5′-TTG ACA AGA ATC CTC ACA ATA CC-3′) and antisense primer Real-SR (positions 309–328, 5′-GGA GGT TGG GGA CTG CGA AT-3′). The quantitative PCR assay was conducted using the commercial SensiFAST SYBR Lo-ROX kit (BIOLINE, London, UK) and primers specific to the S gene with an ABI7500 system (Applied Biosystems, CA, USA).

### Southern Blot Assay

Purification of HBV DNA from the cell pellet was accomplished using the modified method as previously described [33]. Briefly, to detect extracellular HBV DNA the supernatants were collected, centrifuged at 1,300 rpm for 3 minutes, and transferred to clean tubes to remove cellular debris. HBV particles were precipitated from the supernatant using polyethylene glycol 8000 (Sigma-Aldrich, MO, USA) as previously described [34]. Viral pellets were re-suspended in PBS, and DNA was extracted with lysis buffer (0.25% SDS, 250 mM Tris-HCl, pH 7.4, and 250 mM EDTA). The purified HBV DNA was resolved in a 1.5% agarose gel, transferred to a nylon membrane (Hybond N+; Amersham, LC, UK) by southern blotting, and hybridized with a 32P-labeled wild-type, full-length HBV DNA probe generated with a random primer DNA labeling kit (TaKaRa, Tokyo, Japan). Autoradiography was performed and analyzed using the BAS 2500 image analyzer (Fuji Photo Film, Tokyo, Japan).

### Hydrodynamic Injection

For the systemic hydrodynamic injection of pHBV1.2X-Wild and pHBV1.2X-X8Del in vivo, we used six-week-old C57BL/6 female mice with weights of 20–25 g (Orientbio, Seoul, Korea). All experiments utilizing animals in this study were approved by the Seoul National University Institutional Animal Care and Use Committee (IACUC number SNU-140116-4), and the experimental animals were housed in the specific-pathogen-free Laboratory Animal Center. A total of 10 μg of plasmids in PBS was injected into the tail vein within 5–8 seconds. The injected volume was 10% of the mouse’s body mass (e.g., 2 ml for a mouse of 20 g). Ten mice were randomly divided into 2 groups: one injected with pHBV1.2X-Wild and the other injected with pHBV1.2X- X8Del (5 mice per group).

### Statistical Analysis and Ethical Approval

The results are expressed as the mean ± SD from three independent experiments. Data showing a normal distribution were analyzed with Student’s t-test, and the Mann-Whitney U test was used when the data were not normally distributed. Differences between categorical variables were analyzed using Fisher’s exact test or a Chi-square test. A p value < 0.05 (two-tailed) was considered to be statistically significant. All experiments including animals in this study were approved by the Seoul National University Institutional Animal Care and Use Committee (IACUC number SNU-140116-4).

## Results

### The Prevalence of HBV DNA-Positive Subjects among Korean Hepatitis B Vaccinated Subjects and Their Clinical Characteristics

To determine the prevalence of occult HBV infection in a Korean vaccinated population, first, the presence of HBV DNA from a total of 87 HBsAg-negative Korean subjects whose HBV vaccination was verified was investigated via nested PCR targeting the entire S ORF. As a result, HBV DNA was detected from 6 subjects (6.9%, 6/87 subjects). Second, the HBV bDNA level of the 6 subjects with positive nested PCR results was analyzed using the Versant HBV DNA Assay version 3.0. All 6 subjects had DNA levels that were higher than the detection limit of 2,000 copies/ml (average of 38,046 copies/ml) (Table 1). The clinical characteristics of the 6 subjects are shown in Table 2. The ALT levels of all six samples were within the normal range [ALT (IU/l) < 41]; moreover, the anti-HBs levels of all subjects but one (SS-7-5) were positive, the HBeAg levels of all subjects but one (SS-3-22) were positive, and the anti-HBe levels of all subjects but one (SS-3-22) showed that the HBeAg-negative serostatus was non-reactive.

**Table 1:** Clinical and virological data from 6 vaccinated subjects demonstrated to be HBV DNA positive by both the nested PCR protocol and VERSANT HBV DNA 3.0 assay.

Samples	Sex	Age (years)	ALT (IU/L)^a^	HBsAg	Anti-HBs^b^	HBeAg	Anti-HBe^c^	HBV bDNA (copies/ml)^d^
**SS-3-22**	M	41	3	negative	reactive	negative	reactive	141465
**SS-4-7**	F	36	7	negative	reactive	positive	non-reactive	5606
**SS-4-15**	M	35	5	negative	reactive	positive	non-reactive	4342
**SS-4-19**	F	25	9	negative	reactive	positive	non-reactive	40152
**SS-7-5**	F	38	23	negative	non-reactive	positive	non-reactive	9374
**SS-9-43**	M	63	7	negative	reactive	positive	non-reactive	27338

^a^The normal range of ALT (IU/L) is less than or equal to 41.

^b^Anti-HBs antibody (IU/L): non-reactive < 10 and reactive ≥ 10.

^c^Anti-HBe antibody (COI): non-reactive > 1.0 and reactive ≤ 1.0.

^d^The detection limit of HBV bDNA probe (copies/ml) is greater than or equal to 2000.

**Table 2:** Mutation patterns within the HBV full-length genome in the 6 vaccinated subjects verified to be HBV DNA-positive.

No.	Samples	Genotype	SHBs	Polymerase	Precore & Core	X region
1	**SS-3-22**	C2	F220L	L575V	-	T106S, **8-nt deletion (396** ^**th**^ **-404** ^**th**^, C-terminal truncation)
2	**SS-4-7**	C2	-	-	-	**8-nt deletion (396** ^**th**^ **-404** ^**th**^, C-terminal truncation)
3	**SS-4-15**	C2	F220L	L575V	-	**8-nt deletion (396** ^**th**^ **-404** ^**th**^, C-terminal truncation)
4	**SS-4-19**	C2	-	-	-	**8-nt deletion (396** ^**th**^ **-404** ^**th**^, C-terminal truncation)
5	**SS-7-5**	C2	-	-	L137I	T106S, **8-nt deletion (396** ^**th**^ **-404** ^**th**^, C-terminal truncation)
6	**SS-9-43**	C2	-	-	-	**8-nt deletion (396** ^**th**^ **-404** ^**th**^, C-terminal truncation)

### Mutation Analysis of Six Vaccine Escape Subjects by HBV Full-Length Genome Analysis

**Fig 2:**
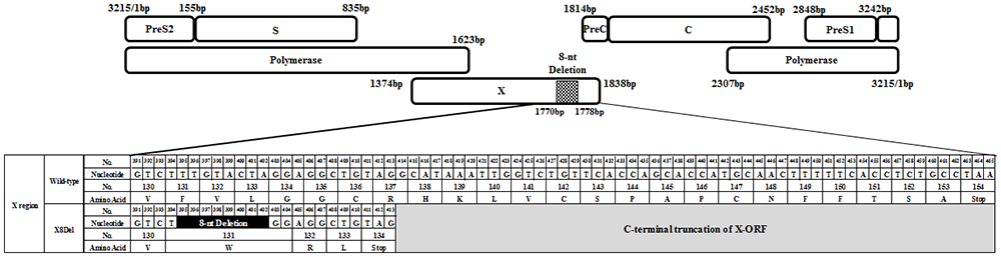
The location of a novel mutation (XDel8) that resulted in the deletion of eight nucleotides (nt 396 to 404) in the C-terminus of the X region found in the 6 vaccinated subjects proven to be HBV DNA positive.

**Fig 3:**
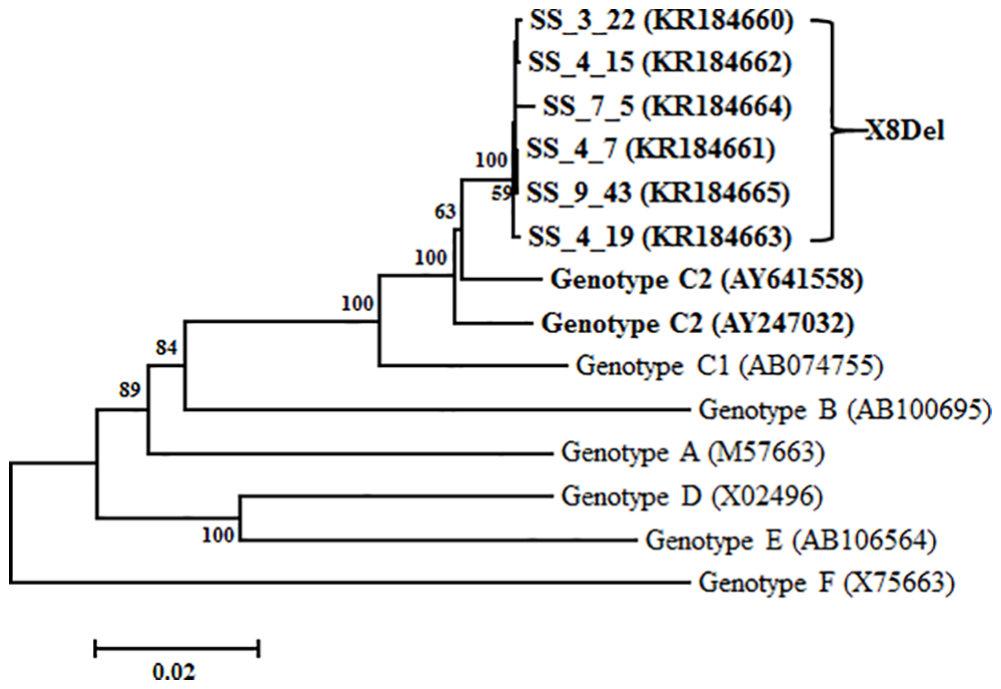
Phylogenetic analysis based on the HBV full-length genome sequences of eight references and the 6 Korean vaccinated subjects proven to be HBV DNA positive. The evolutionary history was inferred using the neighbor-joining method. The percentage of replicate trees in which the associated taxa clustered together in the bootstrap test (1,000 replicates) is shown next to the branches. Phylogenetic analyses were conducted in MEGA4.

To identify mutations related to HBV occult infection from vaccinated individuals, HBV complete genome sequences were analyzed from the 6 subjects with HBV DNA-positive results. To this end, a total of 11 overlapped PCR products covering all of the HBV genome were amplified by nested PCR from 6 subjects and sequenced (Fig 1). Phylogenetic analysis based on complete HBV genome sequences showed that the HBV strains from all 6 subjects belonged to genotype C2 (Fig 2). The mutation patterns of the full-length HBV genome region compared to the reference strains are presented in Table 2. Notably, all 6 subjects had a common mutation type consisting of the deletion of eight nucleotides (nt 396 to 404) in the C-terminus of the X region (hereafter, XDel8) (Fig 3). Moreover, the F220L mutation in the small surface region (HBsAg) that leads to the L575V mutation in the same overlapping polymerase region was found in two samples (SS-3-22 and SS-4-15). Finally, the T106S mutation in the HBV X region (HBxAg) was detected in two samples (SS-3-22 and SS-7-5) (Table 2). In the current study, we focused on X8Del as a novel mutation leading to occult infection in Korean vaccinated subjects because it was present in all of the HBV DNA-positive vaccine subjects.

### Comparison of the HBsAg Secretion and Virion Formation Capacities between the HBV Full-Length Genome Constructs pHBV1.2X-Wild and pHBV1.2X-X8Del in HuH-7.5

**Fig 4:**
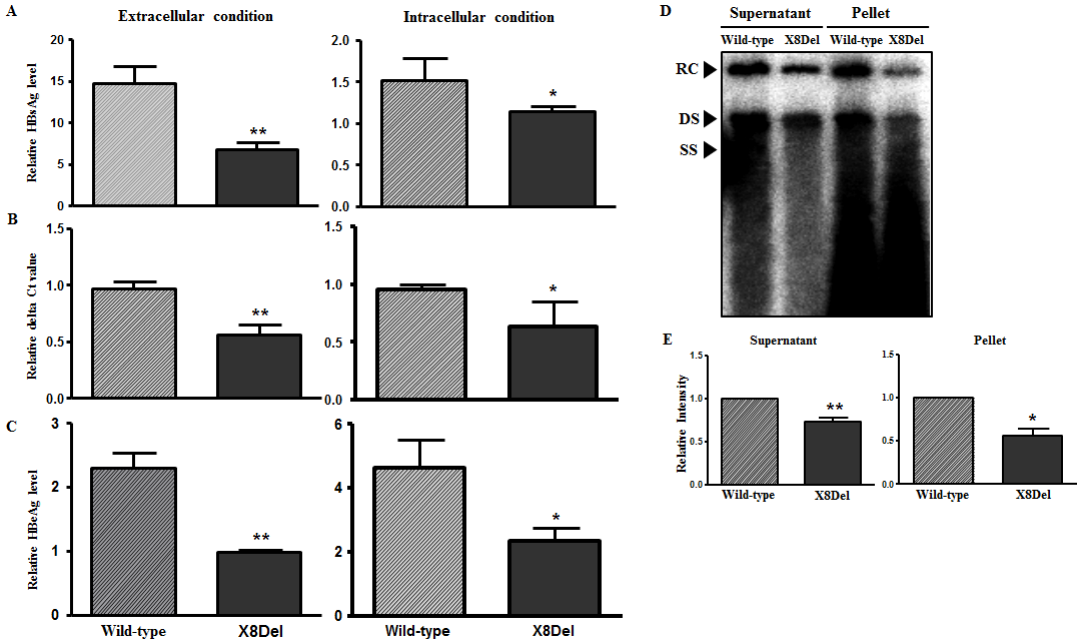
Comparison of the HBsAg secretion and virion formation capacities between the HBV full-length genome constructs pHBV1.2X-Wild and pHBV1.2X-X8Del in HuH-7.5 cells. After the transient transfection of pHBV-1.2x-Wild type and pHBV1.2X-X8Del into HuH-7.5 cells for 2 days, the levels of HBsAg (A) and HBeAg (C) from the supernatant and the RLB-lysed pellet were measured using the HBsAg and HBeAg ELISA kits. The virion levels in the supernatant and pellet after the transfection of both plasmids were compared using real-time quantitative PCR (B) and Southern blotting analysis (D). The relative intensity of DNA from the Southern blotting was measured using ImageJ software (NIH, USA) (E).

To elucidate the occult HBV infection-related mechanism of the X8Del mutation, first, we generated an HBV C2 genotype full-length genome mutant construct harboring the 8-nt deletion in HBxAg, called pHBV1.2X-X8Del, via site-directed mutagenesis of the wild-type full-genome construct pHBV1.2X-Wild. This mutation resulted in a C-terminally truncated HBxAg. Then, we compared the HBsAg and HBeAg secretion and virion formation capacities between the 2 HBV full-length genome constructs (pHBV1.2X-Wild and pHBV1.2X-X8Del) after transient transfection into HuH-7.5 cells. Our ELISA analysis indicated that pHBV1.2X-X8Del exhibited a relatively reduced secretion capacity for HBsAg and HBeAg compared with pHBV1.2X-Wild (Fig 4A and 4C). Our real-time PCR-based analysis for HBV virion quantification indicated that secreted virions from pHBV1.2X-X8Del were also significantly reduced compared with pHBV1.2X-Wild (Fig 4B). A similar result was verified through Southern blot analysis (Fig 4D and 4E). These findings suggest that the major mechanism of X8Del related to occult infection may be the reduced secretion of HBsAg and reduced HBV virion levels.

### Comparison of the HBsAg Secretion and Virion Formation Capacities between the HBV Full-Length Genome Constructs pHBV1.2X-Wild and pHBV1.2X-X8Del in a Hydrodynamic Injection Model

**Fig 5:**
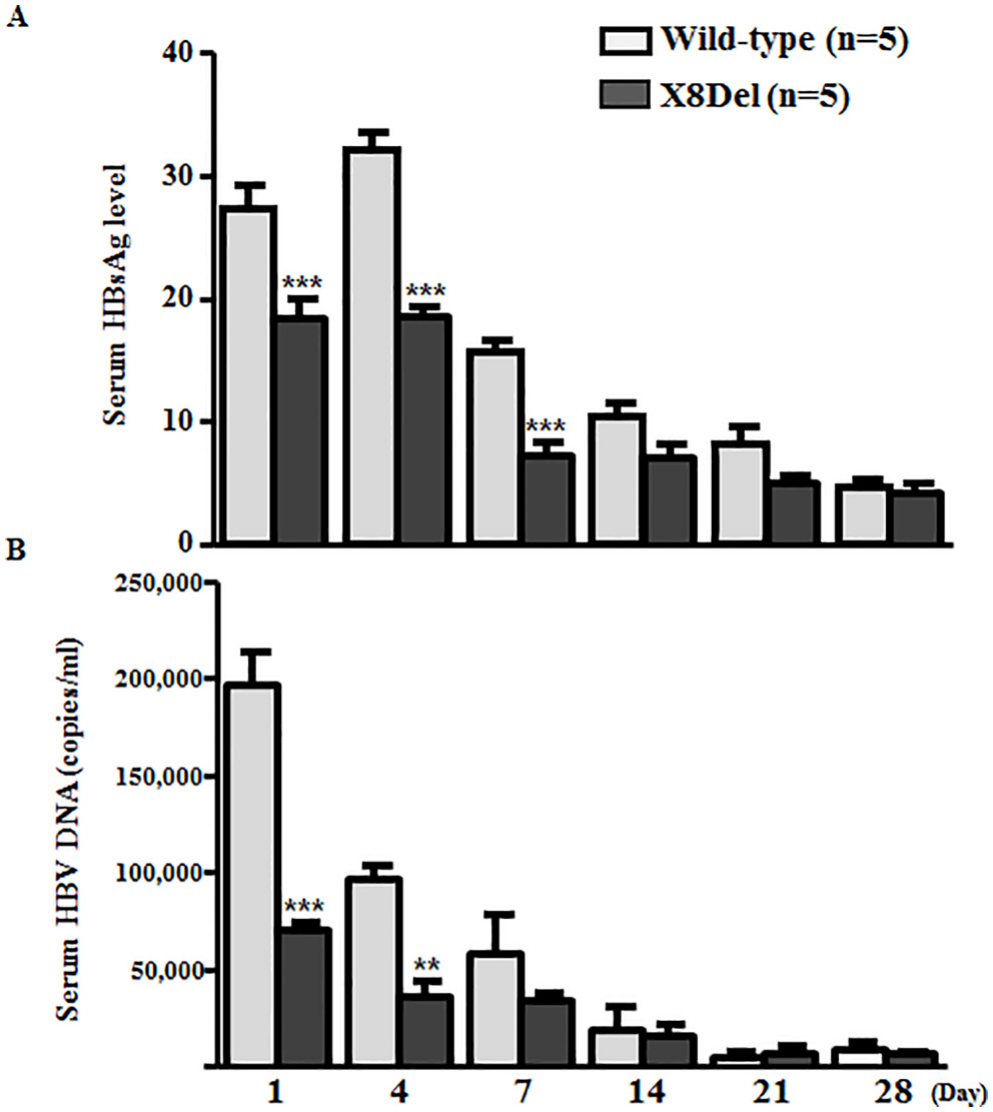
Comparison of HBsAg secretion (A) and virion formation (B) capacities between the HBV full-length genome constructs pHBV1.2X-Wild and pHBV1.2X-X8Del in the hydrodynamic mouse model. Circulating HBsAg from mouse serum was measured using the HBsAg ELISA kit. HBV DNA from virions was measured by real-time quantitative PCR.

To evaluate the effect of X8Del on the secretion of HBV virions and HBsAg in an in vivo mouse model, hydrodynamic tail vein injection of both full-length genome constructs (pHBV1.2X-Wild and pHBV1.2X-X8Del) was applied to deliver the target plasmid to the liver of the mice. Then, secreted HBsAg and virion levels were compared for both plasmids. The HBsAg and viral HBV DNA levels were measured from the mouse serum via orbital sinus blood collection on days 1, 4, 7, 14, 21, and 28. The levels of HBsAg from the serum of mice injected with pHBV1.2X-X8Del were lower compared to mice injected with pHBV1.2X-Wild on days 1, 4, and 7 after injection (Fig 5A). Moreover, viral DNA levels were reduced by approximately three-fold in pHBV1.2X-X8Del compared with pHBV1.2X-Wild at the first time point; this trend was also observed 14 days after infection despite the fact that the contrast between the two plasmids gradually decreased (Fig 5B). These results support the effect of X8Del on the reduction of secreted HBsAg and HBV virion levels.

## Discussion

To date, studies on HBV immune escape mechanisms have mainly focused on mutations within the HBsAg region, particularly within the “a” determinant region; one example is the G145R mutation, which is a major target of HBV serological diagnosis and preventive vaccination [6,7]. However, genotype C2 infection is extraordinarily prevalent in South Korea [18]; thus, atypical mutation patterns in addition to the “a” determinant in the preS/S region are also frequently found in subjects with occult infections [26,35]. Furthermore, our recent study showed that the major mechanism underlying occult infection in South Korea may be attributed to the deficiency in the HBsAg secretion capacity due to mutations outside of the major hydrophilic region (MHR) region rather than changes in Ag-Ab affinity due to “a” determinant mutations [31]. The current study demonstrated a novel HBV genotype C2 mutation type, X8Del. This mutation was an 8-bp deletion in HBxAg that resulted in a C-terminal truncation of this protein. This mutation was detected in 6 Korean vaccinated subjects (Table 2 and Fig 2). Our in vitro (Fig 4) and in vivo experiments (Fig 5) showed negative effects of X8Del on the secretion of HBsAg and HBV virions. These results suggest that one of major mechanisms contributing to vaccine escape in South Korea may be the deficiency of the HBsAg secretion capacity, as shown in the occult infection cases [26,31,35]. To the best of our knowledge, this is the first report showing that an HBxAg mutation contributes to HBV occult infections or vaccine escape.

Notably, all 6 occult infection cases from Korean vaccinated subjects who were positive for HBV DNA replication had a common X8Del type. Our serological analysis of all 6 cases further supports X8Del as a mutation associated with occult infection or vaccine escape. First, despite their HBsAg-negative serostatus, all of the cases showed HBV DNA replication levels that were higher than the detection limit of the HBV bDNA probe (2,000 copies/ml), with an average of 38,046 copies/ml. Furthermore, 5 cases (with the one exception showing HBeAb seroconversion) were HBeAg positive, further indicating that HBV variants of the X8Del type may have typical traits of HBV occult infections: HBsAg seronegative but HBV DNA positive in the serum. Second, anti-HBsAg was detected in 5 cases, suggesting that X8Del may represent a bona fide immune escape mutation (Table 2).

HBxAg has been the focus of a great deal of attention in the HBV field because it has been strongly implicated in hepatocarcinogenesis. HBxAg has a length of 465 bp and encodes a 154-amino-acid protein with an N-terminal negative regulatory domain and a C-terminal transactivation domain. The HBx protein is multifunctional and affects gene transcription, signaling pathways, genotoxic stress responses, cell-cycle control, and apoptosis. HBx also plays an essential role in viral replication [36–38]. Specific mutations in the HBx gene were reported to be related to severe forms of liver disease, such as liver cirrhosis and/or HCC [19,39,40]. Of these, deletions leading to C terminal truncations have been frequently detected in tissues and serum samples in HCC patients. In South Korea, diverse types of HBxAg deletions in the C terminal domain were also frequently detected in patients with severe types of liver disease [22]. Given our previous report that C-terminally deleted HBxAg was frequently found in Korean chronic patients with severe types of liver disease [22], it is tempting to speculate that variants of X8Del induced by host immune pressure from Korean chronic patients may potentially be the infection source for the vaccinated population. This issue remains to be addressed in a future study.

The C-terminal region in HBx has been reported to play a key role in controlling cell proliferation, viability, and transformation [41,42]. Therefore, C-terminally deleted HBx has reduced transactivation activity and inhibitory effects on cell proliferation and thus may contribute to HCC generation. Moreover, its reduced transacting capacity might lead to reduced HBV replication. The results of our in vitro and in vivo study (Figs 4 and 5) are in agreement with a previous result that that a HBV mutant unable to make HBxAg would result in low HBsAg and low viral load in acute phase in a hydrodynamic injection-based C57BL/6 mouse model, suggesting that the loss of HBxAg function may contribute to the higher HBV persistence rate [43]. Interestingly, our clinical data indicated that all 6 subjects proved to have occult infections showed HBsAg negative serostatuses, despite most (5 of 6 subjects) were positive for HBeAg (Table 1). It may be due to the compromising transacting capacity of HBxAg induced by X8Del mutation, which may contribute to modify the overall immune responses to HBV antigens during the course of chronic infections. But, this issue remains to be elucidated in the future study.

To address the issue of whether the reduced secreted HBsAg and HBV virion level of X8Del is from trans-acting mutated HBxAg or cis-acting deleted sequences, co-transfection of both full genome pHBV1.2X construct disrupting start codon of and one of 2 types of sub-genome plasmids, expressing X8Del or wild type HBxAg into HuH-7.5 cells were also done. The similar trend as shown in full genome transfection experiment was also found in co-transfection of both full genome and sub-genome (data not shown). It suggests that modified transacting HBxAg activity of X8Del may lead to reduced HBsAg and HBV virion level, resultantly contributing vaccine escape of X8Del.

In conclusion, our data suggest that a novel vaccine escape mutation, X8Del may contribute to HBV vaccine escape via reduced HBsAg and virion levels that may compromise the HBxAg transacting capacity. To the best of our knowledge, this is the first HBxAg mutation associated with HBV vaccine escape.

## Supporting Information

S1 Table Primers used for the HBV full-genome sequence analysis in this study. (DOC)
