# Supplementary material for: Discovery of a Novel Mutation (X8Del) Resulting in an 8-bp Deletion in the Hepatitis B Virus X Gene Associated with Occult Infection in Korean Vaccinated Individuals
Source: PLoS One. 2015 Oct 5;10(10):e0139551. doi: 10.1371/journal.pone.0139551 (PMC4593592; doi:10.1371/journal.pone.0139551)
Supplement: S1 Table — (DOC) [file pone.0139551.s001.doc]

**Supporting Information**

**S1 Table. Primers used for the HBV full-genome sequence analysis in this study.**
